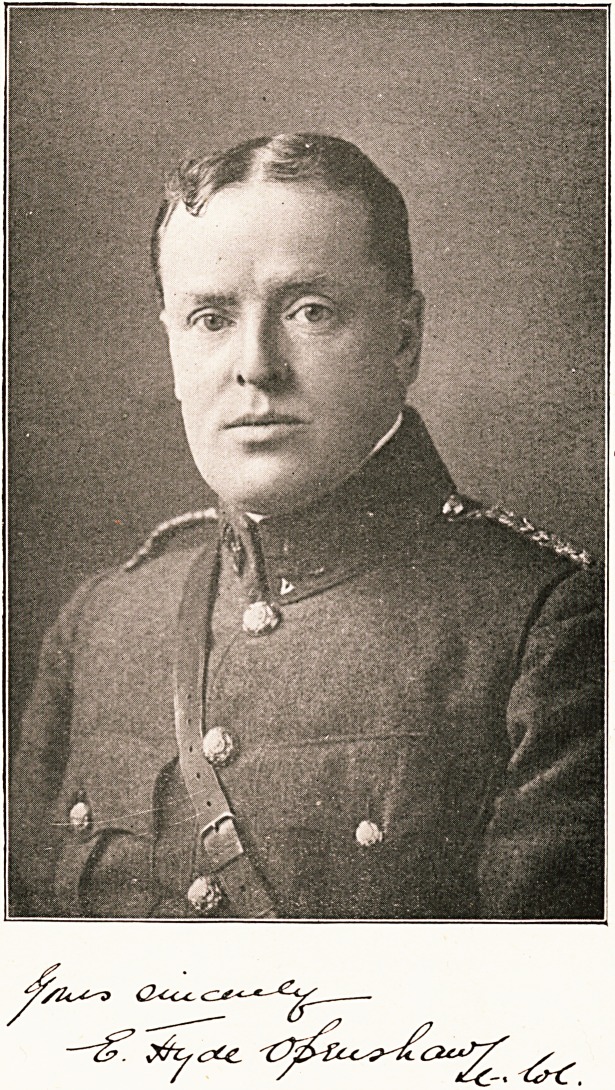# Lieut.-Col. E. H. Openshaw

**Published:** 1917-07

**Authors:** 


### LIEUT.-COL. E. H. OPENSHAW.

In the year 1885 there came to the Bristol Medical School a boyish student, whose bright, happy face and keen, frank eyes reflected a particularly attractive and warm-hearted personality, which none of his teachers or fellow-students can have failed to cherish in memory. So full of life, energetic in work and play alike, thus was Ernest Hyde Openshaw born in the profession he adorned in after years, till the opportunity came with the declaration of war to play a glorious part as a combatant officer for which years of work and preparatory training had fitted him so well. Beloved in his practice and filling an important position in and around Cheddar, many in similar circumstances would have "carried on" at home; but not Openshaw, who was on Salisbury Plain in camp at the outbreak of war, and volunteered at once for service abroad, and sailed as second in command of the 1/4th Somersets for India on October 7th, 1914. His son, E. R. Openshaw, now attached to the Royal Flying Corps, accompanied him as 2nd-Lieutenant in his battalion, and served under him in India and in Mesopotamia. He spent some time at Wellington, in South India, and at Julindur with the battalion, and saw fighting on the N.W. Frontier in the spring of 1916. Ordered to Mesopotamia, the battalion was engaged in the Kut Relief Expeditionary Force, and in the Battle of Dujailah on March 8th he led his men in the assault on the Turkish position. After the hardships of that campaign he was invalided to India, and was later made Commandant of the Convalescent Depot at Wellington, in South India.

He was, however, anxious to return to active service with his battalion, and pressed the authorities to send him back to Mesopotamia. He remained in good health until the beginning of July, when he was reported dangerously ill, and died of heatstroke and heart-failure in hospital at Nasariyeh on the Euphrates at the age of forty-nine.

Colonel Openshaw was the eldest son of the Rev. T. W. Openshaw, for many years Mathematical Master at the Bristol Grammar School, and married the only child of Mr. Harry Fussell, of Bristol. For some time he was House Surgeon of the Bristol Eye Hospital. He went to Cheddar just after his marriage, twenty-three years ago. He was also a prominent Freemason, W.M. of Agriculture Lodge in 1903, and held the provincial rank of Junior Grand Warden of Somerset.

After completing his studies at the Bristol Medical School, Edward Hyde Openshaw qualified as L.R.C.P. and M.R.C.S. in 1890, and after spending his first three years as an assistant at Moretonhampstead and Olveston, joined Dr. R. W. Statham as partner at Cheddar, where he worked until he obeyed his country's call in 1914.

To none are the lines more truly applicable—

"No easy hopes or lies
Shall bring us to our goal;
But iron sacrifice
Of body, will, and soul.
There is one task for all,
For each one life to give.
Who stands if Freedom fall?
Who dies if England live?"

We are glad to give a few words of appreciation from Dr. Statham of the man as he was known by his large circle of friends during a period covering a quarter of a century: "To know Openshaw, or 'Bud,' the name by which he was better known to his intimate friends, was to love him. He was a man of immense power of strength, but with a heart brimful of affection, and as gentle as a child, a type of old English gentleman which we trust may never die out. A thorough sportsman in all its branches, a fearless rider, and in his early days in the football field his name was one to conjure with, he always had a happy smile and was of the most cheerful disposition, usually singing or whistling as he drove along the country lanes, while his hearty laugh and breezy manner won for him the regard and affection of all who came in contact with him. As a doctor he always inspired confidence and hope, and gave ungrudging attention both to rich and poor, possessed of a skill which is seldom met with amongst ordinary practitioners. Many a poor home will miss his generosity, and all his friends will recall those pleasant evenings spent in the garden he loved so well, and which was so wonderful for the unique collection of trees and flowers he had reared and trained with so much pride. He died as he had lived, a man and a true patriot, doing his duty for his country and his fellow-beings."

Like thousands of our fellows, Openshaw has deliberately risked and given all for the sake of those of us who live on, without thought or hope of reward, and in the full belief that we are worthy of the sacrifice. May we who stay safely at home in our tents to "mind the stuff" prove worthy of those who go forth to return beggared in health and strength or never to return. The life and the death of Openshaw is an epic that his widow, children, and friends will always remember with a legitimate pride and reverent love, that will assuage the pain of to-day, and in due time from a grave watered with such tears will spring forth fair flowers to gladden and to inspire afresh their aching hearts.

## Figures and Tables

**Figure f1:**